# Blood ammonia levels in liver cirrhosis: a clue for the presence of portosystemic collateral veins

**DOI:** 10.1186/1471-230X-9-21

**Published:** 2009-03-17

**Authors:** Giovanni Tarantino, Vincenzo Citro, Pasquale Esposito, Sabrina Giaquinto, Annalisa de Leone, Graziella Milan, Francesca Saveria Tripodi, Michele Cirillo, Roberto Lobello

**Affiliations:** 1 Department of Clinical and Experimental Medicine, Hepatology in Internal Medicine Section, Federico II University Medical School of Naples, Naples, Italy; 2 Hepatology Unit of General Medicine, Mauro Scarlato Hospital, Scafati, ASL SA/1, Naples, Italy; 3 Department of Gastroenterology and Digestive Endoscopy, Second University of Naples (SUN), Naples, Italy; 4 Geriatric Centre Frullone ASL NA/1, Naples, Italy; 5 Department of Oncology and Endocrinology, Gastrointestinal Surgery Unit, Federico II University Medical School of Naples, Naples, Italy

## Abstract

**Background:**

Portal hypertension leads to the formation of portosystemic collateral veins in liver cirrhosis. The resulting shunting is responsible for the development of portosystemic encephalopathy. Although ammonia plays a certain role in determining portosystemic encephalopathy, the venous ammonia level has not been found to correlate with the presence or severity of this entity. So, it has become partially obsolete. Realizing the need for non-invasive markers mirroring the presence of esophageal varices in order to reduce the number of endoscopy screening, we came back to determine whether there was a correlation between blood ammonia concentrations and the detection of portosystemic collateral veins, also evaluating splenomegaly, hypersplenism (thrombocytopenia) and the severity of liver cirrhosis.

**Methods:**

One hundred and fifty three consecutive patients with hepatic cirrhosis of various etiologies were recruited to participate in endoscopic and ultrasonography screening for the presence of portosystemic collaterals mostly esophageal varices, but also portal hypertensive gastropathy and large spontaneous shunts.

**Results:**

Based on Child-Pugh classification, the median level of blood ammonia was 45 mcM/L in 64 patients belonging to class A, 66 mcM/L in 66 patients of class B and 108 mcM/L in 23 patients of class C respectively (p < 0.001).

The grade of esophageal varices was concordant with venous ammonia levels (rho 0.43, p < 0.001). The best area under the curve was given by ammonia concentrations, i, e., 0.78, when comparing areas of ammonia levels, platelet count and spleen longitudinal diameter at ultrasonography. Ammonia levels predicted hepatic decompensation and ascites presence (Odds Ratio 1.018, p < 0.001).

**Conclusion:**

Identifying cirrhotic patients with high blood ammonia concentrations could be clinically useful, as high levels would lead to suspicion of being in presence of collaterals, in clinical practice of esophageal varices, and pinpoint those patients requiring closer follow-up and endoscopic screening.

## Background

Portal hypertension leads to the formation of portosystemic collateral veins, of which Esophageal Varices (EV) have the greatest clinical impact and the most severe complications. Specifically, they are discovered on endoscopy in up to two thirds of decompensated cirrhotics. The possibility of identifying cirrhotic patients with EV or other collateral presence by non-invasive means is appealing, in that it could decrease the necessity of endoscopic screening with reduced healthcare costs. The acceptability of such markers depends mainly on their false-negative rate, i.e., those patients with EV and increased risk of bleeding who are not detected because of exclusion from endoscopic screening. Increased spleen volume is an independent predictor of large EV in liver cirrhosis [1], although, a study by Burton et al. casts some doubt on its validity [2]. So far, several studies addressing this issue have been performed with varying success. They have either been based on laboratory parameters, i.e. platelets count (PLTS) or UltraSonographic (US) features [3-10], of which the most interesting seems to be the Spleen Longitudinal Diameter (SLD). Other manifestations of portal hypertension include Portal Hypertensive Gastropathy (PHG), [11] and Large Spontaneous Shunts (LSS). The prevalence of PHG is high (60–80%), but much less in mild portal hypertension (22%), [12]. About 8% of the upper digestive haemorrhages in the cirrhotic patients are secondary to PHG. LSS refer to the presence of patent paraumbilical vein, Spleno-Renal Shunt (SRS), ano-rectosigmoid varices with or without Portal Hypertensive Colopathy (PHC). The prevalence of umbilical vein patency ranges from 6% to 30% in patients with portal hypertension [13]. SRS are present in up to 21% of cirrhotics [14]. Rectosigmoid varices are present in nearly one third of cirrhotic patients, 4% of whom have a lower gastrointestinal hemorrhage, [15]. LSS have been shown to be responsible for recurrent or persistent Portal-Systemic Encephalopathy (PSE), [16]. Actually, ammonia (NH4) levels cannot serve as a laboratory marker for PSE, being neither specific nor highly sensitive [17], although there may be a correlation with severity [18]. The diagnosis of PSE is mainly clinical (neuropsychiatric), and may be confirmed by instrumental measures such as EEG and psychometric testing. This begs the question of whether the routine testing of ammonia levels should be abandoned.

The aim of our study was to investigate the diagnostic utility of venous ammonia levels, spleen size and platelet count as non-invasive markers of shunt presence, with particular emphasis on esophageal varices, and eventual hepatic decompesation. We attempted to correlate these markers with endoscopy findings, US features and Child-Pugh classification as indices of shunt presence (reference tests).

## Methods

### Population

The study included 201 consecutive patients with liver cirrhosis admitted to two Liver Units of university/primary hospitals in Southern Italy, between the period from October 2004 to June 2007 who fulfilled the following criteria: patients' willingness to undergo previously established screening; endoscopic, US and laboratory examinations performed within four weeks of each other; prospective follow-up for a minimum period of 6 months.

Of initial patients, 26 were kept out because their US and laboratory examinations had been previously performed in different centres. Fourteen patients, who had undergone endoscopic esophageal variceal ligation therapy, and eight who had received beta-blockers before US imaging, were also excluded from the study because prior treatment might have caused a change in lesion features.

The remaining 153 patients formed the study population (85 males) whose age ranged from 31 to 85 years (median age 66 years). Chronic liver damage in these patients was caused by hepatitis B (n = 9), hepatitis C (n = 114), alcohol abuse (n = 20) or unknown etiology, likely NonAlcoholic Steato Hepatitis (NASH), (n = 8). Ninety two patients had compensated cirrhosis of the liver. For 121 patients, the diagnosis of cirrhosis was established by contextual clinical (spider nevi, organomegaly) laboratory (low total cholesterol and pseudocholinesterase levels, reduced white blood cell count, globulin/albumin ratio > 1), antecedent imaging data and for 32 patients by biopsy. The non-invasive assessment of liver cirrhosis was blindly performed *de novo *to all patients by radiologists on the basis of US/US-doppler examinations (coarse echo-texture, nodularity presence, increased caudate/right lobe ratio, hypertrophy of the left lobe, characterized by a rounded inferior marginal edge, and portal vein enlargement with decreased flow velocity, absence of a normal doppler waveform, hepatofugal flow). No evidence of hepatocellular carcinoma at the first hepatic decompensation was detected. Renal insufficiency was properly excluded.

### Outcome measures

We assessed the reliability of venous ammonia levels, SLD based on US, and platelet count in predicting the presence of collateral veins, i.e., EV, PHG, LSS including ano-rectal varices, PHC, umbilical vein patency and SRS evidenced by endoscopic and US features.

#### Endoscopic features

##### Esophageal Varices

Esophageal varices were graded according to a previous classification, i.e., F1, small and straight F2, moderately sized, tortuous, and occupying less than one third of the lumen F3, large, coiled, and occupying one third or more of the lumen [19].

##### Portal Hypertensive Gastropathy

PHG was assessed according to the NIEC classification [20]. Mosaic-like pattern was characterized by the presence of small, polygonal areas surrounded by a whitish-yellow depressed border. Red-point lesions were small, flat, lesions (1 mm in diameter). Cherry-red spots were red-coloured, round lesions, slightly protruding into the lumen of the stomach, (2 mm in diameter). Black-brown spots were irregularly shaped flat spots, black or brown, persistently present after washing.

##### Ano-rectal varices and Portal Hypertensive Colopathy

For ethical reasons, only patients who presented with a history of hematochezia at hospital entry underwent colonoscopy to track ano-rectal varices to differentiate from emorroides.

Portal hypertensive colopathy was defined endoscopically in patients with vascular ectasia (solitary or diffuse), redness, and blue vein [21].

##### Hepatic encephalopathy grade

Hepatic encephalopathy was graded based on the level of consciousness, intellectual functions, behaviour and neuromuscular functions according to West Haven (W-H) criteria [22]. W-H grade 0 encephalopathy was ascertained as previously described [23].

##### Assessment for decompesated liver cirrhosis

All patients underwent clinical/US and laboratory assessment to establish the liver cirrhosis severity, using a modified Child-Pugh's classification (Table 1) [24]. Liver decompesation criterion was set at score 8 of Child-Pugh's classification [25].

**Table 1:** Child-Pugh classification slightly modified.

Clinic and laboratory data	Points for increasing abnormality^1^		
	1	2	3
	
**HE (grade^2^)**	None	SHE (0), 1–2	3–4
**Ascites**	None at US	Mild or controlled by diuretics	Present despite diuretics
**PT (% of activity)^3^**	> 70	70–40	< 40
**Albumin (g/dL)**	> 3.5	2.8–3.5	< 2.8
**Bilirubin (mg/dL)**	< 2	2–3	> 3

^**1**^Scoring system: 5–6 points, grade A; 7–9 points, grade B; 10–15 points, grade C. ^**2**^HE: hepatic encephalopathy; Grade 0: sub-clinical hepatic encephalopathy, SHE, unravelled by Retain tests A or B; Grade 1: anxiety, irritability, depression, impaired concentration, sleep disturbances; Grade 2: disorientation, poor short-term memory, disinhibited behavior, drowsiness; Grade 3: somnolence, bizarre behavior, confusion, amnesia, paranoia; Grade 4: Coma. ^**3**^PT, prothrombin time, also expressed in seconds prolonged < 4; 4–6; > 6, or as INR < 1.7; 1.7–2.3; > 2.3. US: ultra sound

##### Venous ammonia concentration

Venous ammonia levels were quantified according to enzymatic determination with glutamate dehydrogenase using rapid and interference-free photometry (340 nm), and were expressed as mcM/L.

Due to reasons of safety, blood was kept chilled and immediately sent to the laboratory for determination.

#### UltraSound features

Spleen measurements of Spleen Longitudinal Diameter (SLD) were performed by postero-lateral scanning with the probe footprint aligned along an intercostal space to provide a longitudinal view of the spleen. The patients were asked to breathe slowly, taking long breaths – as varying degrees of inspiration and expiration are needed to optimize splenic visualization – and to roll on the right side to some extent to aid visualization. At this point, the maximum length, i.e., the optically greatest overall longitudinal dimension obtained from one of the two poles was recorded. Given the high variability in detecting spleen measurements, an US value ≥ 150 mm was chosen to correspond to splenomegaly.

#### Other portosystemic collaterals

Umbilical vein patency, i.e. diameter ≥ 3 mm, was easily detected within the echogenic ligamentum teres hepatis and was confirmed by color Doppler US.

SRS was detected by the same method. The direction of blood flow in the collateral vein was also analyzed in each patient. The Doppler angle used to examine the portal vein was less than 50° in all examinations.

#### Ascites presence

When a patient is in a supine position, free fluid tends to accumulate in the flanks, particularly the superior end of the right paracolic gutter, and in the pelvis due to the effects of gravity. These areas were carefully assessed. Small quantities were sought for around the liver or spleen surface and in the Morrison's pouch [26].

US also helped determine the presence of peritoneo-pleural communications.

### Statistical Analysis

Variables were expressed as median and range. To analyze data regarding the Child-Pugh classification groups, Kruskal-Wallis test was adopted. To better define the pairwise comparison, data were log-transformed and managed by the Student-Newman-Keuls test. The chi square was used to look for differences in the classification system and when stratification was needed multiway tables were generated. Tracking the degree of association between single parameters, i.e., NH4, ALT, Platelets, EV grade and SLD, Spearman's rho for non uniform intervals was used. The relationship between NH4 levels and the W-H grades of PSE and the eventual prediction was studied by linear regression. When confronted with the question of how accurate a parameter was in identifying portosystemic collateral veins presence, the discrimination with relative cut-off or criterion was evaluated using Receiver Operating characteristic Curve (ROC) analysis, graphically expressed as Area Under the ROC (AUROC). Sensitivity (true positive rate), specificity (true negative rate) were also weighted for the same purpose. Optimal cut-off was considered the threshold value with the best specificity/sensitivity.

To predict the presence of portosystemic collateral veins, hepatic decompesation and ascites presence the logistic regression (Enter Method) was employed utilizing as independent variables US values for SLD, platelets count and blood NH4 concentrations. The same tool was carried out to predict the large (II and III grade) EV presence by NH4 concentrations. To assess the independent effect of a quantitative variable, i.e., NH4, on the prediction of the PSE grades linear regression analysis was used. The Factor Analysis was applied to select a subset of variables from a larger set, based on which original variables have the highest correlations with the principal component factors. The Cattel Scree plot, with relative eigenvalues, was performed to screen the real factors. Extraction of the main components amounted to a variance maximizing (varimax) rotation of the original variable space. To assess uniformity, serial measurements were performed, and the data were summarized into two measures, such as: difference last-first value (%) and Area Under the Curve (AUC), creating a clustered multiple comparison graph for the observations.

<Bookmark(4) The concordance correlation coefficient (ρ_c_), [27], which measures precision and accuracy, was adopted to evaluate the degree of pair observations at US in a reduced sample of 21 patients. Statistical analysis was performed operating on Systat 12 and MedCalc Version 9.4 software packages.

This study was based on physicians' adhesion to common diagnostic criteria as previously described and was conducted according to guidelines of the International Conference on Harmonization for Good Clinical Practice, in accordance with the Code of Ethics of the World Medical Association (Declaration of Helsinki).

## Results

The presence of collaterals based on etiology and Child-Pugh's classification is shown in Table 2.

**Table 2:** Collaterals tabulated on aetiology and Child-Pugh classification.

Post-viral	EV	EV	EV	PHG	PHG	PHG	LSS	LSS	LSS
**CHILD**	no	yes	Total	no	yes	Total	no	yes	Total
**A**	40	18	58	23	35	58	56	2	58
**B**	27	33	60	22	38	60	54	6	60
**C**	1	6	7	2	5	7	5	2	7
**Total**	68	57	125	47	78	125	115	10	125
									
**Ethanol**									

**CHILD**	no	yes	Total	no	yes	Total	no	yes	Total
**A**	3	1	4	3	1	4	4	0	4
**B**	6	6	12	8	4	12	9	3	12
**C**	0	4	4	2	2	4	4	0	4
**Total**	9	11	20	13	7	20	17	3	20
									
**Cryptogenic**									

**CHILD**	no	yes	Total	no	yes	Total	no	yes	Total
**A**	2	1	3	3	1	3	2	1	3
**B**	4	1	5	4	1	5	4	1	5
**C**	0	0	0	0	0	0	0	0	0
**Total**	6	2	8	5	3	8	6	2	8

Esophageal Varices (EV), Portal Hypertensive Gastropathy (PHG), Large Spontaneous Shunts, LSS; Pearson Chi-square: 23.672, p = 0.166.

EV was present in 70 patients (45% of the whole population); PHG in 88 cirrhotics (57.5%) and 53 patients had both EV and PHG (34.7%), while LSS in 15 cirrhotics (9.8%), without difference for the aetiology, Pearson Chi-square: 23.672, p = 0.166.

When categorized by Child-Pugh class, splenomegaly, present in 50 subjects (32.7%), showed quite similar distribution in relation to the severity of EV, Table 3.

**Table 3:** Splenomegaly tabulated on severity of esophageal variceal and liver cirrhosis.

Child-Pugh Class A					
**EV grade**	0	1	2	3	Total
**Splenomegaly -**	39	7	4	0	50
**Splenomegaly +**	6	4	3	2	15
**Total**	45	11	7	2	65
					
**Child-Pugh Class B**					

**Splenomegaly -**	27	10	5	6	48
**Splenomegaly +**	10	10	5	4	29
**Total**	37	20	10	10	77
					
**Child-Pugh Class C**					

**Splenomegaly -**	1	1	2	1	5
**Splenomegaly +**	0	2	3	1	6
**Total**	1	3	5	2	11

Splenomegaly absence/presence (rows) by EV, Esophageal Varices grade (columns); Pearson's chi square: 202, p = 0.20. Splenomegaly was detected by measuring SLD, Spleen Longitudinal Diameter at US, with a value set at ≥ 150 mm.

Data of main outcome measures are expresses in Table 4.

**Table 4:** Main outcome measures tabulated on liver cirrhosis severity.

	Child-Pugh Class											
	
	A				B				C			
	
	N	Median	Minimum	Maximum	N	Median	Minimum	Maximum	N	Median	Minimum	Maximum
**NH4** mcM/L	64	45	19	183	66	66	31	221	23	108	28	258
**PLTs** mmc/mL	64	115,000	42,000	293,000	66	86,400	30,000	312,000	23	62,250	27,600	137,000
**SLD** mm	64	133	103	205	66	146	110	212	23	159	100	210

**Figure 1:**
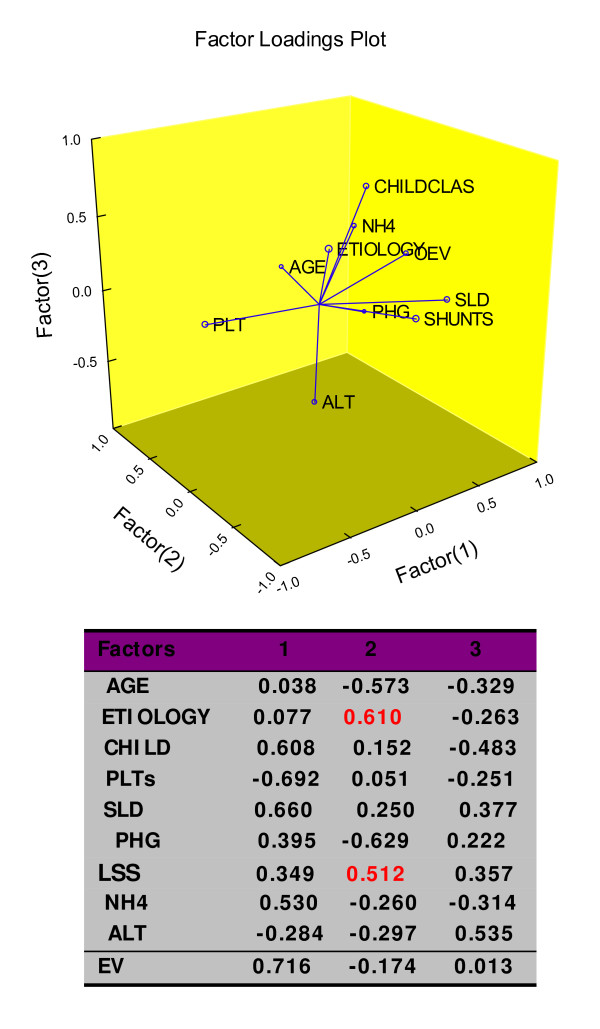
**Hidden relationships unravelled by the Factor Analysis**. Percent of Total Variance Explained by factors: 1 (24.4); 2 (16.3); NH4, Ammonia; PLTs, Platelets count; SLD, Spleen Longitudinal Diameter; EV, Esophageal Varices; PHG, Portal Hypertensive Gastropathy; LSS, Large Spontaneous Shunts; ALT, ALanin Transferase; the critical value was calculated by the formula: Pearson's correlation coefficient for 1% level of significance/square root of patients minus 2, i.e., 419.

NH4 Ammonia; PLTs, Platelets count; SLD, Spleen Longitudinal Diameter. NH4 best differentiated groups, see Figure 1; PLTS differentiated only group A from B, p = 0.013; SLD differentiated only Group A from B, p = 0.24

NH4 levels in A Child-Pugh group were significantly different from the ones of group B and C, i.e., 45 mcM/L in 64 patients belonging to class A, 66 mcM/L in 66 patients of class B and 108 mcM/L in 23 patients of class C respectively, p < 0.001, Student-Newman-Keuls.

The W-H grade 0 was detected in 39.8% of our population. Ascites was present in 35 out of 61 decompesated patients (in one of them hydrothorax was displayed) and in two out of 92 compensed patients (chi square = 61, p < 0.001).

### Associations

First of all, EV grade was well correlated with NH4 levels, i.e., rho 0.43, p < 0.001, explaining the 18.5% of variance. A significant correlation was evidenced between SLD values and EV grades, i.e. rho = 0.35, p < 0.0001. The continued analysis of blood NH4 concentrations highlighted a significant (p = 0.02) correlation between them and platelets count, i.e. -0.18, although explaining only the 3.2% of variance; vice versa, no correlation existed with ALT, i.e., 0.14 (p = 0.06) nor with SLD, i.e., 0.10 (p = 0.2).

An inverse correlation, i.e., rho -0.53 (p = < 0.0001) was detected between SLD and PLTS count. By detecting the structure in the relationships among variables (Factor Analysis), the EV presence showed the highest value, i.e., 0.716 followed by the classic sign of portal hypertension, i.e., splenomegaly at US and by the severity of liver cirrhosis, weighted as Child-Pug's classification, but not the PHG evidence, Figure 1. In the same setting, interestingly, LSS were correlated to the liver cirrhosis aetiology, being more frequent, but not statistically different, in cryptogenic form (chi square for trend 3.1, p = 0.07).

### Accuracy and prediction

**Figure 2:**
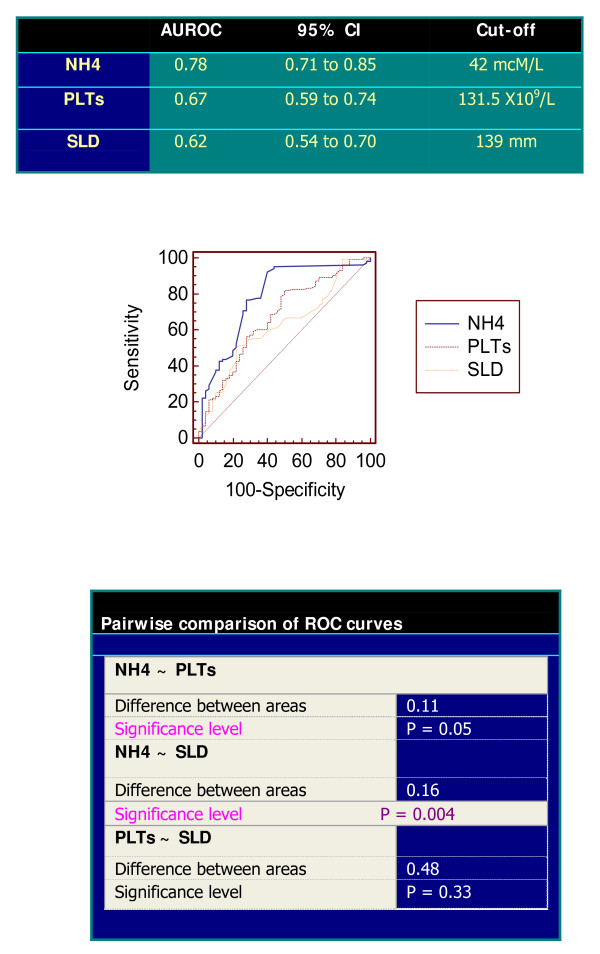
**Comparison of ROC curves using as classification variable the presence of Collaterals**. NH4, Ammonia; PLTs, Platelets count; SLD, Spleen Longitudinal Diameter; NH4 cut-off (42 mcM/L) had sensibility of 92% and specificity of 60%, with 8% of false negative results; CI, Confidence Intervals.

When comparing ROC curves to classify the collaterals presence, NH4 levels gave the best AUROC among PLTS count and SLD at US, significantly superior to the other ones. The AUROC of NH4 in predicting the EV presence alone resulted quite similar to that predicting all the shunts, i.e., 0.75 (95% CI 0.68–0.82; sensitivity 97%, specificity 43% with the same criterion of 42 mcM/L) versus 0.78, Figure 2. As matter of fact, two out of 70 patients, with normal NH4 concentrations (28 and 42 mcM/L, respectively), having certain EV presence, would be excluded by the endoscopic performance and only one out 36 patients would have been missed having large varices if this threshold had been used. The false positive rate at this cut-off was 28.1%, i.e., 43 patients.The AUROCs of NH4 levels and the PLTs/SLD ratio similarly predicted EV existence (0.75 versus 0.73, p = 0.70).

Among the same parameters, ammonia levels predicted hepatic decompensation and ascites presence (Odds Ratio 1.018, 95% C. I. 1.009–1.028, Std. Coef. 4. 04, p < 0.001 and 1.018, 95% C. I. 1.009–1.027, Std. Coef. 3.92, p < 0.001, respectively). At the same manner, blood NH4 concentrations best predicted the demonstration of portosystemic veins, among PLTs and SLD, Table 5. Interestingly, NH4 concentrations gave a clear prediction of large varices (Odds Ratio 1.012, 95% C. I. 1.004–1.020, Std. Coef. 0.012, p < 0.002, with 96.6 and 2.78% of correct diagnosis in absence/presence of their detection (on the whole 74.5%).

**Table 5:** Prediction of Esophageal Varices and collaterals.

a	**95% C. I**.		
**Parameter p-value**	**Odds Ratio**	**Lower**	**Upper**
**NH4 < 0.001**	1.017	1.0075	1.027
**PLTs 0.03**	1.001	1	1.001
**SLD 0.4**	0.985	0.95	1.016
**PLTs/SLD 0.012**	0.99	0.98	0.99.8

**b**	**95% C. I**.		

**Parameter p-value**	Odds Ratio	Lower	Upper
**NH4 < 0.001**	1.023	1.010	1.035
**PLTs 0.085**	0.994	0.986	1.001
**SLD 0.233**	1.011	0.993	1.028

Logistic regression using Esophageal Varices (a) and collaterals (b) presence as dependent variable; NH4 Ammonia; PLTs, Platelets count; SLD, Spleen Longitudinal Diameter; PLTs/SLD, Platelets count Spleen Longitudinal Diameter ratio index.

Ammonia levels did not predict severity of PSE measured as W-H grades, i.e., coefficient = 5.04, beta = 0.088, p = 0.28.

### Uniformity

**Figure 3:**
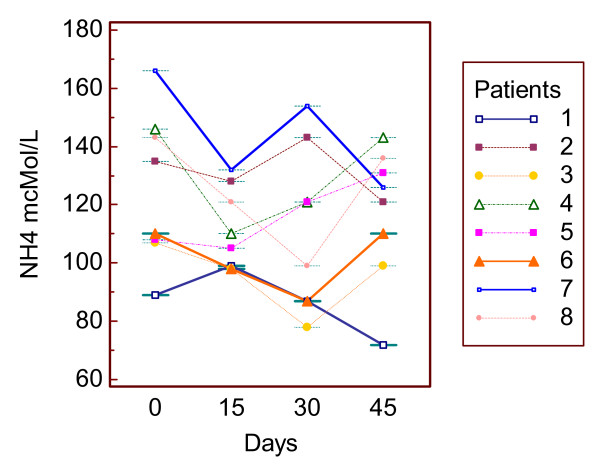
**Serial measurements**. Clustered multiple comparison graph showing a good repeatability of blood ammonia concentration over time; median of the maximum difference versus first value percentage = -22.5, 95% C.I. -29.74 to 12.4; median of the AUC, (baseline = first value) = -577.5, C.I. = -1027.76 to 229.84; AUC, Area Under the Curve.

To asses if blood ammonia concentrations of cirrhotic patients were constant on time, eight of them were studied at a two week-distance for one month and half. The results showed a low difference of the remaining values versus the first one and a near flat AUC, as evidenced in Figure 3.

The agreement of US paired observations ranked high, i.e., ρ_c _= 0.91.

## Discussion

Authorities give emphasis on following facts: j) the issue of identifying patients with EV at risk of bleeding by non invasive means is relevant and has been addressed in several recent studies – this research is yet another attempt to achieve this goal – jj) the timing of endoscopic evaluation is basic because hemorrhage from EV threatens the patient's life.

Commenting on the results, we can observe that our data in cirrhotic patients support a good association between blood NH4 levels and EV presence. As matter of fact, the blood ammonia determination suffers from some limits in its measurements. In fact, the collection, handling, storage, and analysis of blood samples are all potential sources of error. Anyway, recommendations ought to be made on the collection and processing of blood samples, for it is by standardization and rigid adherence to these techniques that the reliability of the test results will be improved. The utility of such a marker as the proposed one relies on easily repeatability, but no way it should substitute endoscopy screening.

Our data somehow agree with the body of present knowledge. In fact, when comparing the performance of blood ammonia with the PLTs/SLD ratio [9], the only marker contextually studied, we found a not so much dissimilar reliability. The advantages of the Plts/SLD ratio are evident because they do not suffer from external confounding factors. The disadvantages are consistent with the fact that thrombocytemia is sometime related to the auto-antibodies presence that turns out in falsely low count of PLTs. Indeed, the opposite, falsely high count of PLTs, could be detected in patients suffering from liver cirrhosis with hepatocarcinoma.

We think that the key point is not whether to recommend endoscopy or not but when to undergo it. Decision about the optimal intervals for surveillance mainly to detect large varices depends on what proportion of patients that bleeds before starting prophylactic treatment we are willing to accept. If we set the acceptable level of risk at 10%, patients with no varices at baseline can be re-endoscoped at 3-year intervals; for patients with small varices at baseline, the recommended interval of 1–2 years [27] R. de Franchis, Updating consensus in portal hypertension: report of the Baveno III consensus workshop on definitions, methodology and therapeutic strategies in portal hypertension, J. Hepatol. **33 **(2000), pp. 846–852. should be maintained [28]. According to our results, patients with high levels of NH4 should undergo endoscopy faster.

As preliminary remark, we admit that in determining portal hypertension, mechanisms potentially reversible are involved, i.e., contractility of sinusoidal lining cells, systemic mediators of arteriolar resistance, production of endothelins or nitric oxide, and swelling of hepatocytes [29]. However, other irreversible factors such as tissue fibrosis and regeneration increase resistance in the sinusoids and terminal portal venules, playing a key role. Even though PHG is recognized as a clinical entity associated to portal hypertension, its significance has not yet been elucidated [30].

Discussing possible mechanisms and explanations for our findings, it is noteworthy to stress the fact that portal hypertension contributes slowly but constantly to hepatic insufficiency. Our observation that blood NH4 levels predicted both ascites and collaterals presence is intriguing, reinforcing the concept of a common origin. Data from long follow-up of patients suffering from compensated cirrhosis B show that poor hepatic reserve and severity of portal hypertension significantly correlate [31]. At the same manner, measurements of portal pressure provide unique prognostic information for predicting portal hypertensive-related bleeding and mortality in patients with alcoholic cirrhosis [32], also in patients without clear presence of EV [33]. High values of blood NH4 are important because they point out an incoming liver decompesation; in fact, less blood reaches the liver, diminishing thus the hepatic reserve. Portal-systemic collaterals provide a pathophysiological route to decompress the hypertensive portal system. Despite this, the vascular resistance of the collateral bed is still greater than the resistance of the liver, and portal pressure does not decrease [34]. What is more, ammonium compounds increase vascular tone by causing influx of extracellular calcium through the voltage-dependent calcium channel and intracellular alkalinisation [35]. The opposite effect (depleting intracellular Ca2+ stores on flow-induced vascular dilatation) was proved in rat isolated small mesenteric arteries [36].

Examining the limitations of the present study we have to pinpoint that as indirect evidence of portosystemic shunts presence, some laboratory parameters have already been proposed, i.e., Serum Bile Acids (SBA) and Indocyanine Green Clearance (IGC). Portosystemic shunting in dogs occurs frequently either as congenital anomalies of the portal vein or as acquired shunting due to portal hypertension secondary to parenchymal liver disease or portal vein thrombosis. In these animals, the two most commonly used screening tests for detecting shunts are SBA and plasma ammonia concentrations. Both are reliable, but blood ammonia level is a higher sensitive and specific parameter [37]. Following this evidence, we might have dosed the SBA levels, to confirm our hypothesis, and probably post-prandial rather than fasting SBA values, but this test is subject to some limitations. In fact, impaired hepatic uptake could be the major determinant of the rise in SBA levels in chronic liver disease [38]. Secondly, high SBA values have been reported in patients with small bowel colonisation [39]; abnormal SBA concentrations have been described in hyperlipidaemia [40], and changing SBA status has been noted with alteration of the dietary vitamin C [41]. IGC, measured by percentage disappearance rate, detects alterations in liver function [42]. Given that these disappearance rates (percentage) have been found to correlate with Child's-Pugh scores in a series of cirrhotic patients [43], it is likely that blood ammonia levels and IGC provide similar assessments of the severity of hepatic decompesation.

Further drawbacks to our study could be unreported variation of diet (hyperproteic) by patients and missed occult blood loss. Our repeated measure of blood NH4 did not significantly change, thus minimizing the possibility of hyperproteic diet influence on ammonia levels. A further, more important, disadvantage could be represented by the temporary use of non-absorbable disaccharides by 18 out of 23 decompesated patients. In fact, although there is some criticism about their being of benefit to lower NH4 [44], they are still considered standard treatment for PSE. Clearly, Child's-Pugh classification is not studied as a risk factor for shunting, because internal and external validity may be compromised as some predictors may not be independent of each other or of outcomes. Colonoscopy was not offered to every patient. Finally, although detection of other portosystemic collaterals besides EV improves the specificity of NH4, it is barely important in clinical practice as prophylactic remedies are not warranted. On the contrary, our figures are corroborated by previous data speculating on hyperintense globus pallidus on T1-weighted cranial magnetic resonance imaging [45].

## Conclusion

Identifying cirrhotic patients having high ammonia levels could be useful, as they lead the treating physician to suspect an eventual presence of collaterals, mainly esophageal varices. This would encourage the use of endoscopy screening in this at-risk population, and increase the physicians' surveillance for ascites and/or PSE. Although some data are expected, this study is the first to demonstrate on a sufficiently large scale the strict correlation between NH4 levels and portosystemic collateral channels that carry blood away from the portal venous system to the general circulation. But, are these efforts worth to? In fact, for some Authorities, in primary prophylaxis of variceal bleeding, as first strategy, patients should be given a beta-blocker without undergoing upper endoscopy [46]. Anyway, this issue still remains unsolved because this practice is associated with increased adverse effects, such as hypotension, and minimal reduction in mortality.

## Abbreviations

EV: Esophageal Varices; PHG: Portal Hypertensive Gastropathy; PHC: Portal Hypertensive Colopathy; LSS: Large Spontaneous Shunts; SRS: Spleno-Renal Shunt; PSE: Portal-Systemic Encephalopathy; NH4: Ammonia; W-H: West Haven; SLD: Spleen Longitudinal Diameter; US: UltraSound; ROC: Receiver Operating characteristic Curve; AUROC: Area Under the ROC; AUC: Area Under the Curve; PLTS: Platelets; IGC: Indocyanin Green clearance; SBA: Serum Bile Acids.

## Competing interests

The authors declare that they have no competing interests.

## Authors' contributions

VC, PE, SG, AdL, GM, FST MC and RL carried out the clinical and instrumental investigation. VC participated in its design and coordination. GT conceived of the study, drafted the manuscript and performed the statistical analysis. All authors read and approved the final manuscript.

## Pre-publication history

The pre-publication history for this paper can be accessed here:

http://www.biomedcentral.com/1471-230X/9/21/prepub
